# *Cissus Quadrangularis* enhances *UCP1* mRNA*,* indicative of white adipocyte browning and decreases central obesity in humans in a randomized trial

**DOI:** 10.1038/s41598-021-81606-9

**Published:** 2021-01-21

**Authors:** Saimai Chatree, Chantacha Sitticharoon, Pailin Maikaew, Kitchaya Pongwattanapakin, Issarawan Keadkraichaiwat, Malika Churintaraphan, Chanakarn Sripong, Rungnapa Sririwichitchai, Sompol Tapechum

**Affiliations:** Department of Physiology, Faculty of Medicine Siriraj Hospital, Mahidol University, 2 Wanglang Rd., Siriraj, Bangkoknoi, Bangkok, 10700 Thailand

**Keywords:** Hormones, Endocrinology, Endocrine system and metabolic diseases, Medical research

## Abstract

Obesity is associated with the growth and expansion of adipocytes which could be decreased via several mechanisms. *Cissus Quadrangularis* (CQ) extract has been shown to reduce obesity in humans; however, its effect on human white adipocytes (hWA) has not been elucidated. This study aimed to investigate the effects of CQ on obesity, lipolysis, and browning of hWA. CQ treatment in obese humans significantly decreased waist circumference at week 4 and week 8 when compared with the baseline values (*p* < 0.05 all) and significantly decreased hip circumference at week 8 when compared with the baseline and week 4 values (*p* < 0.05 all). Serum leptin levels of the CQ-treated group were significantly higher at week 8 compared to baseline levels (*p* < 0.05). In hWA, glycerol release was reduced in the CQ-treated group when compared with the vehicle-treated group. In the browning experiment, pioglitazone, the PPAR-γ agonist, increased *UCP1* mRNA when compared to vehicle (*p* < 0.01). Interestingly, 10, 100, and 1000 ng/ml CQ extract treatment on hWA significantly enhanced *UCP1* expression in a dose-dependent manner when compared to pioglitazone treatment (*p* < 0.001 all). In conclusion, CQ decreased waist and hip circumferences in obese humans and enhanced *UCP1* mRNA in hWA suggestive of its action via browning of hWA.

## Introduction

Obesity, a state of excessive fat accumulation leading to weight gain^1^, causes many adverse health consequences including diabetes mellitus^2^, hypertension^3^, and cardiovascular diseases^4^ and was reported to be raised globally between 1980 and 2015^5^. Obesity is related to the growth of adipose tissue leading to increased adipocyte size or hypertrophy and increased adipocyte number or hyperplasia^6^. Hypertrophy is caused by excess accumulation of triglycerides in the cytosol of adipocytes while hyperplasia refers to pre-adipocyte proliferation and differentiation^6^. The decrease in adiposity is revealed via several mechanisms including the reductions in pre-adipocyte proliferation and differentiation, the increases in adipocyte lipolysis and apoptosis^7,8^, and induction of browning of white adipocytes leading to increased thermogenesis^9^.

Lipolysis is the breakdown of triglycerides into glycerol and free fatty acids (FFAs) which are released into the circulation^10^. Browning of white adipocytes is the transformation of white adipocytes to the new type of adipocytes known as brown-like adipocytes or beige/brite adipocytes^11^. The transformation results in a change from unilocular morphology of white adipocytes to multilocular morphology of brown adipocytes and increased expression of uncoupling protein 1 (UCP1) which is the marker of brown adipocytes^12,13^. UCP1 in brown adipocytes produces heat for thermoregulation^14^, thereby increasing the body’s energy expenditure.

*Cissus Quadrangularis* (CQ) is a medicinal herb commonly used in India, Africa^15^, and Thailand^16,17^ for a century. The bioactive component of CQ is composed of hexadecanoic acid, n-hexadecanoic acid, bis (2-methylpropyl) ester, 1, 2-benzenedicarboxylic acid, phytol, ethylester, caffeine, and dibutyl phthalate^18,19^. CQ is normally used for healing of broken bones and injured ligaments and tendons, analgesic, antioxidant^15^, anti-inflammation^20^, and relief of hemorrhoidal symptoms^16^. Moreover, it has benefits in weight reduction and improvement of metabolic syndrome^21^. The study in obese and overweight Cameroonians showed that supplementation of 300 mg/day CQ extract (CQR-300) with no dietary restriction for 8 weeks decreased body weight, waist circumference, blood glucose levels, LDL cholesterol levels but not body fat percentage when compared to the baseline values^22^. Treatment with combination of CQR-300 and Irvingia gabonensis extracts had greater reduction of those parameters than treatment with the CQR-300 alone^22^. Another study in Cameroonian subjects showed that administration of 300 mg daily of a proprietary extract of CQR-300 for 6 weeks with a 2100 kcal/day diet resulted in reduced total cholesterol and triglycerides in obese and overweight humans when compared to baseline values^23^. However, studies in humans were performed in only 1 institution and the underlying mechanism of CQ extract on mature adipocytes has not been elucidated. This study aimed to 1) study the effects of CQ extract on obesity reduction and the improvement of metabolic parameters in obese human subjects, 2) investigate the effect of CQ extract on human adipocyte lipolysis, and 3) investigate the effect of CQ extract on browning of human white adipocytes.

## Results

### The effects of CQ extract on obesity and clinical parameters in obese subjects

**Figure 1:**
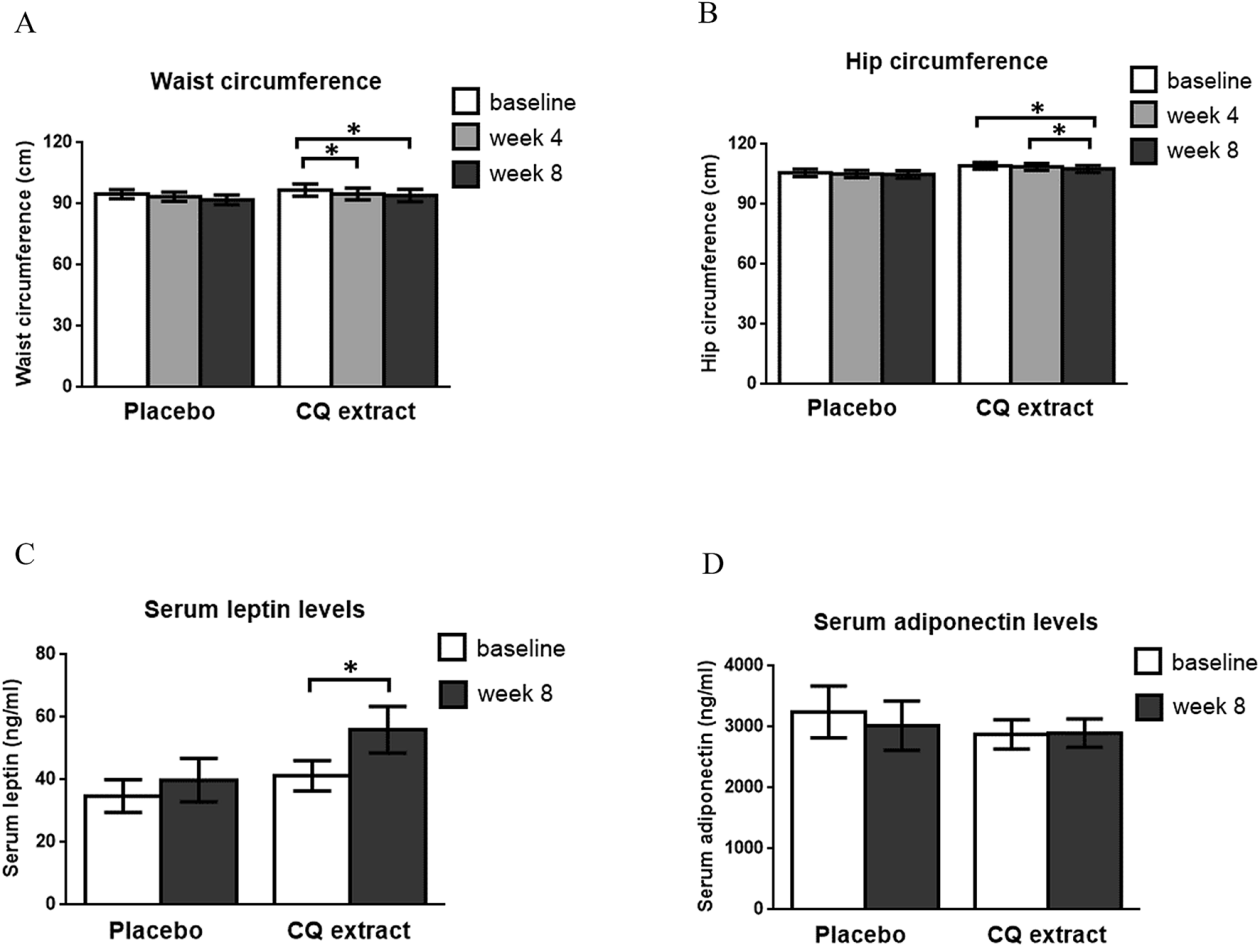
Mean (± SEM) waist circumference (panel **A**), hip circumference (panel **B**), serum leptin levels (panel **C**), and serum adiponectin levels (panel **D**) before and after supplement with placebo or CQ extract in obese human subjects. *p < 0.05, n = 15/group.

The effects of CQ extract on obesity and clinical parameters in obese subjects are shown in Table 1 and Fig. 1A, B. From the two-way mixed-design analysis of variance (ANOVA), there were no effects of treatments or time points or interaction between treatments and time points on body weight [(F = 1.17, *p* = 0.289), (F = 0.50, *p* = 0.610), and (F = 0.29, *p* = 0.750), respectively]; BMI [(F = 0.19, *p* = 0.668), (F = 0.67, *p* = 0.515), and (F = 0.32, *p* = 0.727), respectively]; total body fat percentage [(F = 0.01, *p* = 0.920), (F = 0.33, *p* = 0.720), and (F = 0.46, *p* = 0.633), respectively]; total body fat mass [(F = 0.61, *p* = 0.443), (F = 0.31, *p* = 0.733), and (F = 0.28, *p* = 0.756), respectively]; systolic blood pressure (SBP) [(F = 0.01, *p* = 0.923), (F = 0.04, *p* = 0.962), and (F = 1.96, *p* = 0.150), respectively]; and diastolic blood pressure (DBP) [(F = 0.031, *p* = 0.862), (F = 0.51, *p* = 0.603), and (F = 0.55, *p* = 0.583), respectively].

**Table 1:** The effects of CQ extract on obesity and clinical parameters in obese subjects.

	Placebo			CQ extract		
	baseline	Week 4	Week 8	baseline	Week 4	Week 8
Age (years)	36.27 ± 2.39			32.40 ± 1.96		
Body weight (kg)	76.43 ± 3.65	76.79 ± 3.64	76.58 ± 3.72	82.27 ± 3.77	82.33 ± 3.75	82.37 ± 3.96
BMI (kg/m^2^)	29.38 ± 0.94	29.53 ± 0.94	29.39 ± 0.95	30.00 ± 0.90	30.03 ± 0.89	30.01 ± 0.94
Total body fat percentage (%)	37.51 ± 1.92	37.75 ± 1.96	36.95 ± 1.96	37.59 ± 1.77	37.66 ± 1.53	37.71 ± 1.67
Total body fat mass (kg)	28.94 ± 2.40	29.07 ± 2.37	28.57 ± 2.38	31.44 ± 1.77	31.06 ± 1.91	31.15 ± 2.07
SBP (mmHg)	120.93 ± 3.94	124.20 ± 5.23	124.00 ± 5.54	124.13 ± 2.67	121.73 ± 2.50	121.73 ± 2.34
DBP (mmHg)	81.87 ± 3.09	84.00 ± 3.12	81.73 ± 2.99	82.13 ± 3.22	83.20 ± 3.01	84.20 ± 1.75

Values are expressed as mean ± SEM. n = 15/group.

Remarkably, from the two-way mixed-design ANOVA, there was a significant effect of time points but no effects of treatments or interaction between treatments and time points on waist circumference [(F = 9.32, *p* < 0.001), (F = 2.34, *p* = 0.632), and (F = 0.16, *p* = 0.857), respectively]; and hip circumference [(F = 119.55, *p* < 0.001), (F = 1.21, *p* = 0.281), and (F = 0.36, *p* = 0.697), respectively]. Mean (± SEM) waist circumference of the CQ-treated group was significantly decreased at week 4 (94.83 ± 2.82) and week 8 (94.03 ± 2.99) when compared to the baseline value (96.77 ± 2.95) (*p* < 0.05 all) (Fig. 1A). On the other hand, in the placebo-treated group, mean (± SEM) waist circumference was comparable among at baseline (94.83 ± 2.14), week 4 (93.50 ± 2.20), and week 8 (92.07 ± 2.38) time points (Fig. 1A). The mean (± SEM) hip circumference of the CQ-treated group was significantly decreased at week 8 (107.73 ± 1.73) when compared with baseline (109.23 ± 1.67) and week 4 (108.73 ± 1.69) values (*p* < 0.05 all) (Fig. 1B). In placebo-supplemented subjects, mean (± SEM) hip circumference was not different among at baseline (105.71 ± 1.90), week 4 (105.20 ± 1.76), and week 8 (104.93 ± 1.88) time points (Fig. 1B).

### The effects of CQ extract on peripheral metabolic factors in obese subjects

The effects of CQ extract on peripheral metabolic factors in obese subjects are shown in Table 2. From the two-way mixed-design ANOVA, there was a significant effect of time points (F = 5.81, *p* = 0.023) but had no effects on treatments (F = 1.35, *p* = 0.255) or interaction between treatments and time points (F = 0.38, *p* = 0.542) on fasting plasma glucose levels. Mean (± SEM) fasting plasma glucose levels were comparable between baseline and week 8 of the CQ-treated group but the levels were significantly increased at week 8 when compared with baseline levels (*p* < 0.05) in the placebo-treated group (Table 2). There were no effects of treatments or time points or interaction between treatments and time points on fasting plasma insulin [(F = 0.00, *p* = 0.984), (F = 0.38, *p* = 0.540) and (F = 1.74, *p* = 0.198), respectively]; triglyceride [(F = 1.78, *p* = 0.193), (F = 0.01, *p* = 0.928), and (F = 1.02, *p* = 0.320), respectively]; total cholesterol [(F = 0.11, *p* = 0.741); (F = 0.66, *p* = 0.425); and (F = 0.72, *p* = 0.403), respectively]; high-density lipoprotein (HDL) cholesterol [(F = 2.57, *p* = 0.120), (F = 0.08, *p* = 0.777), and (F = 0.86, *p* = 0.362), respectively]; low-density lipoprotein (LDL) cholesterol [(F = 0.12, *p* = 0.727), (F = 0.54, *p* = 0.468), and (F = 0.21, *p* = 0.653), respectively]; the homeostatic model assessment of insulin resistance (HOMA-IR) [(F = 0.04, *p* = 0.838), (F = 0.09, *p* = 0.771), and (F = 2.05, *p* = 0.163), respectively]; and the quantitative insulin sensitivity check index (QUICKI) [(F = 0.32, *p* = 0.576), (F = 0.07, *p* = 0.801), and (F = 0.84, *p* = 0.368), respectively].

**Table 2:** The effects of CQ extract on peripheral metabolic factors as well as liver and kidney functions in obese subjects.

	Baseline		Week 8	
	Placebo	CQ extract	Placebo	CQ extract
Plasma glucose (mg/dL)	94.53 ± 2.48	92.13 ± 1.70*	98.13 ± 2.43	94.27 ± 1.55
Plasma insulin (μU/mL)	17.38 ± 4.42	20.10 ± 4.31	18.87 ± 3.58	15.95 ± 1.72
HOMA-IR	4.07 ± 1.03	4.47 ± 0.88	4.57 ± 0.87	3.72 ± 0.39
QUICKI	0.33 ± 0.01	0.32 ± 0.07	0.32 ± 0.01	0.32 ± 0.01
Plasma triglyceride (mg/dL)	142.73 ± 22.51	113.20 ± 13.35	153.20 ± 32.39	104.47 ± 12.92
Plasma total cholesterol (mg/dL)	190.20 ± 7.96	189.60 ± 6.77	195.67 ± 8.40	189.47 ± 7.06
Plasma HDL cholesterol (mg/dL)	44.67 ± 2.81	51.20 ± 3.91	43.53 ± 3.08	51.80 ± 3.43
Plasma LDL cholesterol (mg/dL)	117.31 ± 6.63	115.76 ± 6.60	121.48 ± 7.87	116.75 ± 5.83
Total Bilirubin (mg/dL)	0.47 ± 0.04	0.45 ± 0.05	0.45 ± 0.05	0.45 ± 0.05
Direct Bilirubin (mg/dL)	0.16 ± 0.01	0.15 ± 0.02	0.15 ± 0.01	0.15 ± 0.02
AST (U/L)	19.13 ± 1.78	16.80 ± 1.40	20.73 ± 1.61	21.00 ± 2.87
ALT (U/L)	19.93 ± 2.46	19.00 ± 3.66	21.67 ± 3.40	22.93 ± 4.65
BUN (mg/dL)	10.55 ± 0.72	11.85 ± 0.92	11.11 ± 0.83	11.19 ± 0.59
Creatinine (mg/dL)	0.76 ± 0.05	0.80 ± 0.04	0.81 ± 0.05	0.82 ± 0.05
eGFR (ml/min)	100.19 ± 7.19	106.68 ± 3.71	103.44 ± 3.84	104.54 ± 4.45

Values are expressed as mean ± SEM. n = 15/group, *p < 0.05 compared with placebo.

*HOMA-IR* the homeostatic model assessment of insulin resistance, *QUICKI* the quantitative insulin sensitivity check index, *HDL* high-density lipoprotein, *LDL* low-density lipoprotein, *AST* aspartate aminotransferase, *ALT* alanine aminotransferase, *BUN* blood urea nitrogen, *eGFR* estimated glomerular filtration rate.

### The effects of CQ extract on serum leptin and adiponectin levels

The effects of CQ extract on serum leptin and adiponectin levels are shown in Fig. 1C&D, respectively. From the two-way mixed-design ANOVA, there was a significant effect of time points (F = 8.69, *p* = 0.007) but no effects on treatments (F = 1.51, *p* = 0.230) or interaction between treatments and time points (F = 0.94, *p* = 0.341) on serum leptin levels. CQ extract significantly increased leptin levels at week 8 of the supplement when compared to baseline levels (*p* < 0.05) (Fig. 1C). In the placebo-supplemented group, serum leptin levels were not different between week 8 and baseline levels (Fig. 1C). There were no effects of treatments (F = 0.29, *p* = 0.593) or time points (F = 0.47, *p* = 0.500) or interaction between treatments and time points on serum adiponectin levels (F = 0.67, *p* = 0.419).

### The effects of CQ extract on blood chemistry of kidney and liver functions in obese subjects

The effects of CQ extract on blood chemistry of kidney functions including blood urea nitrogen (BUN), creatinine, and estimated glomerular filtration rate (eGFR) and liver functions including total bilirubin, direct bilirubin, aspartate aminotransferase (AST), and alanine aminotransferase (ALT) in obese subjects are shown in Table 2. There were no effects of treatments or time points or interaction between treatments and time points on BUN [(F = 0.49, *p* = 0.490), (F = 0.01, *p* = 0.929), and (F = 1.59, *p* = 0.218), respectively]; creatinine [(F = 0.15, *p* = 0.702), (F = 1.87, *p* = 0.182), and (F = 0.64, *p* = 0.430), respectively]; eGFR [(F = 0.01, *p* = 0.930), (F = 2.43, *p* = 0.130), and (F = 0.13, *p* = 0.723), respectively]; total bilirubin [(F = 0.03, *p* = 0.857), (F = 0.30, 0.587), and (F = 0.07, *p* = 0.794), respectively]; direct bilirubin [(F = 0.02, *p* = 0.900), (F = 0.58, *p* = 0.453), and (F = 0.03, *p* = 0.875), respectively]; AST [(F = 0.19, *p* = 0.664); (F = 3.44, *p* = 0.074); and (F = 0.69, *p* = 0.413), respectively]; and ALT [(F = 0.01, *p* = 0.931); (F = 3.52, *p* = 0.071), and (F = 0.08, *p* = 0.786), respectively].

### The effect of CQ extract on human adipocyte lipolysis

**Figure 2:**
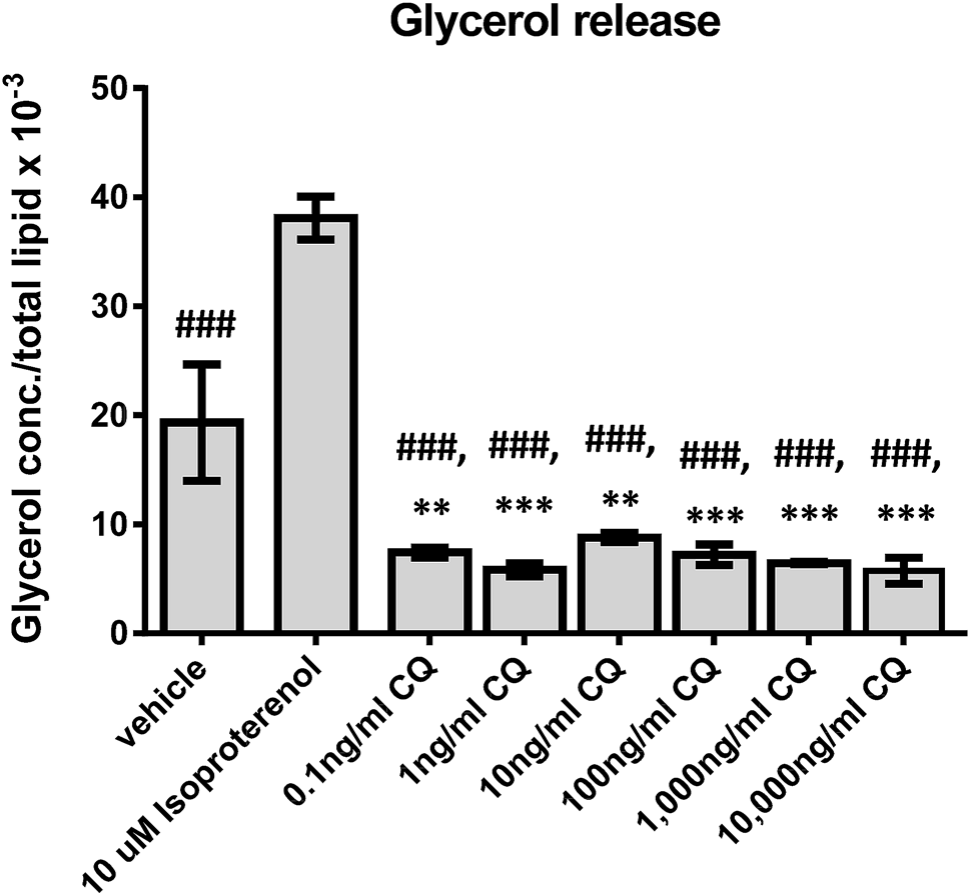
Mean (± SEM) glycerol release determined by glycerol concentrations normalized to total lipid after supplements with difference doses of CQ extract for 24 h, **p < 0.01 compared with vehicle treatment, ^###^*p* < 0.001 compared with isoproterenol treatment.

The effect of CQ extract on human adipocyte lipolysis is shown in Fig. 2. The glycerol release of the isoproterenol-treated group was significantly higher (*p* < 0.001) than the vehicle-treated group and the 0.1, 1, 10, 100, 1000, and 10,000 ng/ml CQ extract-treated groups (Fig. 2). On the other hand, glycerol release of the 0.1, 1, 10, 100, 1000, and 10,000 ng/ml CQ extract-treated groups was significantly lower than that of the vehicle- and isoproterenol-treated groups (*p* < 0.01 all, Fig. 2).

### The effect of CQ extract on browning of human white adipocytes

**Figure 3:**
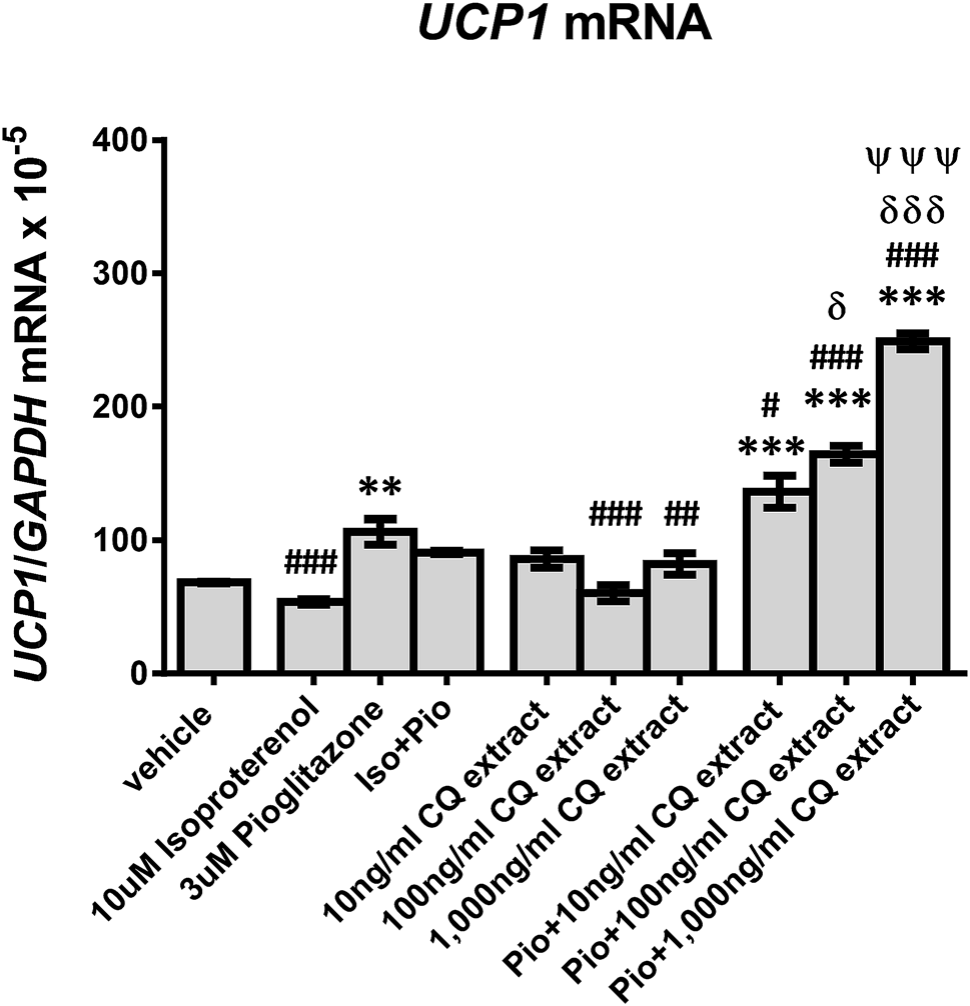
Mean (± SEM) *UCP1* mRNA expression normalized to *GAPDH* (reference gene) in different conditions of treatment for 7 days. **p < 0.01, ***p < 0.001 compared with vehicle treatment, ^#^*p* < 0.05, ^##^*p* < 0.01, ^###^*p* < 0.001 compared with pioglitazone treatment, ^δ^*p* < 0.05, ^δδδ^*p* < 0.001 compared with pioglitazone plus 10 ng/ml CQ extract treatment, ^ψψψ^*p* < 0.001 compared with pioglitazone plus 100 ng/ml CQ extract treatment.

The effect of CQ extract on browning of human white adipocytes is shown in Fig. 3. *UCP1* mRNA was significantly higher in the pioglitazone (*p* < 0.01) and pioglitazone combined with 10, 100, and 1000 ng/ml CQ extract-treated groups (*p* < 0.001 all) when compared with the vehicle- or 10 μM isoproterenol- or 100 ng/ml CQ extract- or 1000 ng/ml CQ extract-treated groups (*p* < 0.01 all) after treatments for 7 days (Fig. 3). *UCP1* mRNA of the isoproterenol-, isoproterenol combined with pioglitazone-, and 10 ng/ml CQ extract-treated groups was comparable with that of the vehicle-treated group (Fig. 3). Interestingly, the 10, 100, and 1000 ng/ml CQ treatments on human white adipocytes significantly enhanced *UCP1* mRNA expression when compared to the pioglitazone treatment alone (*p* < 0.001 all, Fig. 3). Moreover, the 1000 ng/ml CQ extract combined with pioglitazone treatment significantly increased *UCP1* mRNA when compared to the 10 and 100 ng/ml CQ extract combined with pioglitazone treatment (*p* < 0.001, Fig. 3). Also, the 100 ng/ml CQ extract combined with pioglitazone treatment increased *UCP1* mRNA when compared to the 10 ng/ml CQ extract combined with the pioglitazone treatment (*p* < 0.05, Fig. 3).

## Discussion

We found that 300 mg of CQ supplement twice a day (600 mg per day) in obese subjects did not reduce body fat mass or fat percentage which was in accordance with a previous study^22^. Moreover, we found that CQ supplement had no effect on the reduction of body weight or BMI. Our results were inconsistent with the previous study showing that treatment of 150 mg CQ extract twice a day (CQR-300) in overweight and obese subjects (BMI > 26 kg/m^2^) significantly decreased body weight after 4 and 8 weeks of treatment^22^. Although the previous study found a reduction in body weight, body fat percentage was comparable among at baseline, week 4, and week 8 of the treatment^22^. We postulate that the reduction in body weight of the subjects in the previous study^22^ might not be from the reduction in fat mass but might probably be from other components including fat-free mass (skeletal muscle, heart, and other organs), bone minerals, and body water. Furthermore, the different findings might be from different sample sizes, doses of CQ extract, and phases of the menstrual cycle of female subjects. The previous study had 24 subjects per group^22^ while our study had 15 subjects per group. The dose of CQ extract of our study was 600 mg per day while that of the previous study was 300 mg per day^22^. In our study, the experiments in female subjects were performed at the early proliferative phase (Day1–Day3) while the previous study^22^ did not mention the phases of menstrual cycle which might lead to confounding effects of sex steroids on body weight and body fat from different phases of menstrual cycle^24,25^.

Interestingly, we found that the waist circumference of subjects treated with CQ was significantly decreased at week 4 around 1.94 cm (94.83 ± 2.82 cm) and week 8 around 2.74 cm (94.03 ± 2.99 cm) after treatment when compared to the baseline values (96.77 ± 2.95 cm). Our result was in accordance with a previous study showing that CQ extract treatment for 8 weeks significantly decreased waist circumference when compared with the initial values^22^. The effect of CQ on waist circumference reduction might be beneficial for decreases in visceral obesity, risk of metabolic syndrome, and obesity‐related morbidity due to accumulation of abdominal fat^26^. Moreover, the hip circumference of the CQ-treated group was significantly decreased at week 4 around 0.5 cm (108.73 ± 1.69 cm) and at week 8 around 1.5 cm (107.73 ± 1.73 cm) when compared with the baseline values (109.23 ± 1.67 cm). These results suggest that CQ treatment had little effect on total body fat but rather had more impact on central fat reduction.

Our study found that CQ extract did not reduce SBP, DBP, plasma glucose and insulin, HOMA-IR, QUICKI, total cholesterol, triglyceride, LDL cholesterol, HDL cholesterol, and adiponectin levels. These results indicated that CQ extract at dose 300 mg twice a day might not have the effects on metabolic parameters in obese subjects. Although CQ extract was found to decrease waist circumference, the degree of reduction might be too small to impact these parameters. Our results were inconsistent with a previous study showing that total cholesterol, plasma LDL, and blood glucose were reduced after 8 weeks of treatment with daily 300 mg CQ extract in overweight and obese subjects^22^. The different findings might be from the reasons mentioned earlier in paragraph 1.

This study showed that the supplement of 600 mg/day of CQ extract for 8 weeks did not show any adverse effects on liver and kidney functions. The liver chemistry parameters and kidney chemistry parameters were fallen in the normal ranges. These data suggest the safety of CQ usage on liver and kidney functions at the dose of 300 mg twice a day for 8 weeks. CQ-supplemented subjects reported some adverse effects including nausea (n = 1), dizziness (n = 1), headache (n = 1), stomach gas (n = 1), mouth ulcer (n = 1), dry mouth (n = 3), constipation (n = 3), diarrhea (n = 1), and warm sensation (n = 4) while placebo-supplemented subjects reported that they had nausea (n = 1), dizziness (n = 1), warm sensation (n = 1), constipation (n = 1), and dry mouth (n = 2).

Surprisingly, even the subject’s body weight, BMI, and body fat mass and percentage were comparable between before and after CQ treatment, serum leptin levels of CQ-treated subjects were significantly higher at week 8 by 1.29 times when compared with their baseline values. This result suggests that CQ increased leptin levels without changes in adiposity. The underlying mechanism is not known.

We further investigated the effect of CQ extract on lipolysis of human adipocytes. To the best of our knowledge, this is the first study that determined the lipolytic effect of CQ extract. Our results showed that 10 μM isoproterenol (the non-specific β-adrenergic receptor agonist) which was the positive control, significantly increased glycerol release (the index of lipolysis)^27^ into the medium when compared to vehicle suggestive of its lipolytic effect. On the other hand, 0.1, 1, 10, 100, 1000, 10,000 ng/ml CQ extract treatments decreased glycerol release in the medium from human adipocytes suggesting that all doses of CQ extract decreased lipolysis. A previous study found that long term leptin treatment inhibited lipolysis by reducing hormone-sensitive lipase (HSL) expression^28^. Taken together, we hypothesized that CQ-decreased lipolysis might probably be via the action of leptin. The other possible mechanisms of lipolysis reduction of CQ might be via insulin, alpha 2 adrenergic receptor, and adenosine together with their signaling cascades. The underlying mechanism of CQ extract on lipolysis reduction should be further investigated.

Furthermore, we explored the novel mechanism of CQ on adiposity reduction through the browning of human white adipocytes. Our results showed that pioglitazone alone and pioglitazone plus 10 ng/ml, 100 ng/ml, and 1000 ng/ml CQ extract treatments significantly increased *UCP1* mRNA when compared with the vehicle treatment. However, isoproterenol or CQ extract treatment alone did not increase *UCP1* mRNA expression compared to vehicle. These results indicate that peroxisome proliferator-activated receptor-γ (PPAR-γ) is essential for the enhancement of *UCP1* mRNA. UCP1 is the marker of brown adipocytes^12,13^ and its upregulation indicates the thermogenic characteristic of brown adipocytes^14^. It is known that PPARs play a pivotal role in the regulation of transcriptional thermogenic genes^29,30^. Both PPAR-γ and PPAR-α agonists upregulated the expression of brown adipocyte specific genes including *UCP1* and *peroxisome proliferator-activated receptor γ co-activator 1-α* (*PGC1-α*)^30^.

Remarkably, 10, 100, and 1000 ng/ml CQ extract combined with pioglitazone enhanced *UCP1* expression by 1.28, 1.55, and 2.34 times, respectively, when compared to pioglitazone alone. These results suggest that CQ extract might augment the browning effect on human white adipocytes dose-dependently. Interestingly, a recent study showed that leptin directly promoted browning of white adipocytes via Janus kinase 2/signal transducer and activator of transcription 3 signaling pathway^31^. Moreover, a recent study in C57BL/6 mice showed that leptin decreased signaling cascades of the Hedgehog (Hh) signaling pathway^32^. Hh pathway, being recently understood in the last few years, has been shown to regulate proliferation, differentiation, stem cell population, tissue polarity, and, carcinogenesis^33^. The activation of the Hh pathway by enhancement of its transcription factors, *glioma-associated oncogene* (*Gli*) *1/2/3,* was found to inhibit browning of white adipocytes^32^. Leptin was found to inhibit the Hh pathway by decreasing *Gli 1/2/3* resulting in increased white adipocyte browning and upregulated thermogenic genes including *UCP1*, *PGC1-α*, and *PR-domain containing 16*^32^. As we found higher leptin levels in subjects treated with CQ for 8 weeks compared to their baseline levels, we hypothesized that CQ could induce browning of white adipocytes via the action of leptin.

CQ extract could probably be used as a novel agent for obesity treatment via enhancement of browning of white adipocytes leading to increased thermogenesis and thus increased energy expenditure. Although PPAR-γ agonist could increase *UCP1* mRNA and induce white adipocyte browning^11^, it causes weight gain and visceral obesity^34,35^ by its actions through adipocyte differentiation^35^, increased lipid accumulation in mature adipocytes, and inhibition of lipolysis^36^. Thus, the enhancement of *UCP1* mRNA by CQ might augment white adipocyte browning leading to increased thermogenesis and energy expenditure with less adipogenic properties.

Although many studies in mice revealed that browning of adipose tissue was associated with increased thermogenesis and energy expenditure leading to weight loss^37–39^, the browning effect of adipose tissue associated with increased energy expenditure and weight loss in humans is still under debate. After mild cold exposure, human subjects with high brown adipose tissue mass increased daily energy expenditure by 14.7% while subjects with low brown adipose tissue mass slightly increased their energy expenditure by 0.4%^40^. Another study in humans showed that brown adipose tissue mass had a positive correlation with whole-body energy expenditure^41^. However, this study revealed that the brown adipose tissue-specific energy expenditure was not associated with the whole-body energy expenditure and contributed to only almost 1% of total body expenditure rising^41^ suggesting that brown adipose tissue is a minor candidate in cold-induced thermogenesis. Thus, the use of activated brown adipose tissue as a potential strategy to induce weight loss is still inconclusive.

In the human study, CQ extract could not reduce body weight, BMI, body fat mass or body fat percentage. This might be because CQ has both adiposity and anti-adiposity effects via inhibition of lipolysis and induction of white adipocyte browning, respectively. As CQ was shown to decrease waist circumference, one of the metabolic risk factors, it might be considered as a natural agent to improve metabolic syndrome. In addition to the mechanisms discussed above, CQ was shown to decrease adipogenesis^42^ via decreased lipid accumulation and reduced adipogenesis/lipogenesis-related mRNA expression levels of *lipoprotein lipase*, *fatty acid- binding protein*, *fatty acid synthase*, *stearoyl-CoA desaturase-1*, and *acetyl-CoA carboxylase*^42^.

Our study had limitations as the followings: 1) in the human experiment, energy consumption and energy expenditure were not controlled because we aimed to investigate the supplement effects of the substance; 2) the dose and duration of CQ treatment in humans might not be optimal; and 3) the browning of adipocytes has not been confirmed by the histological study.

In conclusion, CQ extract was shown to decrease waist circumference and hip circumference in obese human subjects. We revealed novel findings that CQ treatment increased leptin levels in humans, decreased glycerol release suggestive of its antilipolytic effect, and enhanced *UCP1* mRNA suggestive of increased browning of white adipocytes. However, the effect of CQ extract on adipose tissue remains to be explained. Additional studies are required to disclose the mechanisms involved in CQ induced browning of white adipocytes. The use of CQ on obesity treatment would be a future challenge to augment its effects on adiposity reduction rather than obesity enhancement. Dose, duration, and forms of the application need to be further investigated to get the maximal effect on obesity treatment.

## Methods

### The study in obese human subjects

#### Subjects

**Figure 4:**
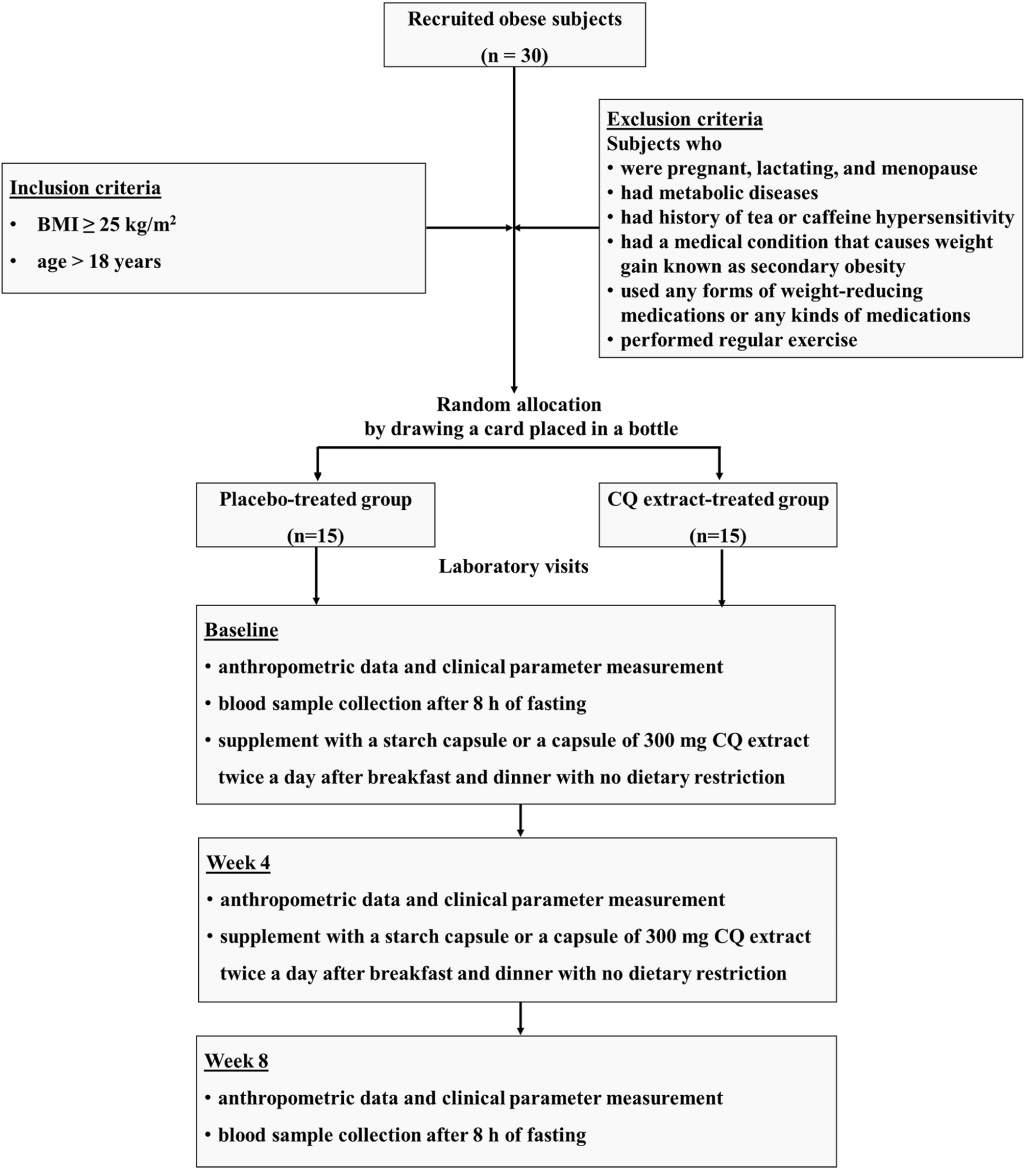
The study protocol of the human experiment.

The study protocol was approved by the Siriraj Institutional Review Board of the Faculty of Medicine Siriraj Hospital, Mahidol University (Si 563/2015) in full compliance with international guidelines for human research protection such as the Declaration of Helsinki, the Belmont Report, CIOMS Guidelines and the International Conference on Harmonization in Good Clinical Practice (ICH-GCP). The research project was approved for registration at Thai Clinical Trials Registry (TCTR) since 2020-04-21 20:31:49 and the TCTR identification number is TCTR20200422001. Informed consents were obtained from all subjects prior to the study. Obesity is classified as body mass index (BMI) ≥ 25 kg/m^2^ for Asian-Pacific populations^43,44^ and BMI ≥ 30 kg/m^2^ for Caucasian populations^45^. Thirty Thai obese subjects, BMI ≥ 25 kg/m^2^ and age > 18 years, were recruited and randomly divided into 2 groups (n = 15/group) including the CQ extract-treated group and the placebo (starch capsule)-treated group. Each subject was randomly assigned to one of the groups by drawing a card placed in a bottle (a total of 15 cards/group). The enrollment of participants and drawing card generation were performed by the researchers. Subjects who were pregnant, lactating, and menopause; had metabolic diseases (e.g. diabetes mellitus, hyper- or hypo-thyroidism, Cushing syndrome), history of tea or caffeine hypersensitivity, and a medical condition that causes weight gain known as secondary obesity (e.g. endocrine disorders, hypothalamic disorders, and some congenital conditions); used any forms of weight-reducing medications or any kinds of medications; and performed regular exercise (at least 30 min/session, 3 times per week^46^) were excluded. The allocation of subjects as well as the inclusion and exclusion criteria are shown in Fig. 4.

### The study protocol

This study was a double-blind, placebo-controlled clinical trial. Subjects in the CQ extract-treated group and placebo-treated group received a capsule of 300 mg CQ extract or a starch capsule, respectively, twice a day after breakfast and dinner for 8 weeks with no dietary restriction. All supplement capsules were identical in appearance and shape with neither subjects nor researchers knowing which supplement received by the participants. All subjects attended laboratory visits at the Department of Physiology, Faculty of Medicine Siriraj Hospital, Mahidol University for 3 time points which were at baseline or week 0, week 4, and week 8 for anthropometric data and clinical assessment. Blood samples were collected after 8 h of fasting at week 0 and week 8. The study protocol is shown in Fig. 4. Female subjects participated in each experiment on the early proliferative phase of their menstrual cycle (Day 1–3) to control the confounding effects of sex steroids while male subjects participated at any day. All subjects were instructed to maintain their normal diet and physical activity patterns during the entire study. Moreover, a designed calendar was given to all subjects to mark off when they took each supplement capsule as well as record their diet 3 days prior to the next visit and the side effects of the intervention. At the end of week 4 and week 8, all subjects had to bring both the medication and calendar for drug compliance checking. There were no withdrawn subjects during the research experiment.

### Demographic and anthropometric data and blood pressure measurement

Data of age, body weight, height, BMI, waist circumference, hip circumference, total body fat percentage, and total body fat mass were collected. Total body fat percentage and total body fat mass were assessed by TANITA, the digital scale for body composition measurement using the bioelectrical impedance method^47^. All subjects were instructed to wear light clothing and stand on the TANITA machine with bare feet by using the 0.3 kg adjustment for the clothing weight. Waist circumference was determined at the umbilicus level in the standing position when subjects performed silent breathing^48^. Hip circumference was determined at the widest part of the buttocks^49^. SBP and DBP were recorded by using a sphygmomanometer after 30-min bed rest in the supine position.

### Hormonal assay

The measurements of blood glucose, insulin, total cholesterol, HDL cholesterol, LDL cholesterol, triglyceride, BUN, creatinine, total bilirubin, direct bilirubin, ALT, and AST were performed by the central laboratory at the Department of Clinical Pathology, Faculty of Medicine Siriraj Hospital, Mahidol University, Thailand. Fasting blood glucose was analyzed by the enzymatic hexokinase method and fasting blood insulin was analyzed by the sandwich immunoassay using electro-chemiluminescence immunoassay (ECLIA). Total, HDL, and LDL cholesterols, triglyceride, and total and direct bilirubin were analyzed by the enzymatic colorimetric method. BUN was analyzed by the urease/glutamate dehydrogenase coupled enzymatic technique. Creatinine was analyzed by the modification of the UV enzymatic method by Oliver and Rosalk. ALT and AST were analyzed by the enzymatic reaction method. Fasting plasma glucose and insulin levels were applied to determine levels of insulin resistance by the HOMA-IR method and insulin sensitivity by the QUICKI method^50^. The HOMA-IR is calculated by the following formula: HOMA-IR = (fasting glucose (mg/dl) x fasting insulin (µU/ml))/405. The QUICKI is calculated from the following formula: QUICKI = 1/((log(fasting insulin (µU/mL)) + log(fasting glucose (mg/dL)).

### Analysis of serum leptin and adiponectin levels

Serum leptin and adiponectin levels were analyzed by the commercial enzyme-linked immunosorbent assay (ELISA) (Phoenix Pharmaceuticals Inc., California, USA) kits according to the manufacturer’s guidelines. The ranges of detection were 0.313–20 ng/ml for leptin and 0.15–10 ng/ml for adiponectin. The minimum detectable concentrations were 0.313 ng/ml for leptin and 0.15 ng/ml for adiponectin. Serum samples were diluted with the assay buffer with 1:5 dilution for leptin and 1:5000 dilution for adiponectin. The intra-assay and inter-assay variations were 6.66% and 6.74%, respectively for leptin, and 2.33% and 3.55%, respectively for adiponectin. The absorbance of optical density (O.D.) was read at 450 nm by the Synergy HT Multi-Detection Microplate Reader (BioTek Instruments, Inc., Vermont, USA).

### The study in human adipocyte culture

**Figure 5:**
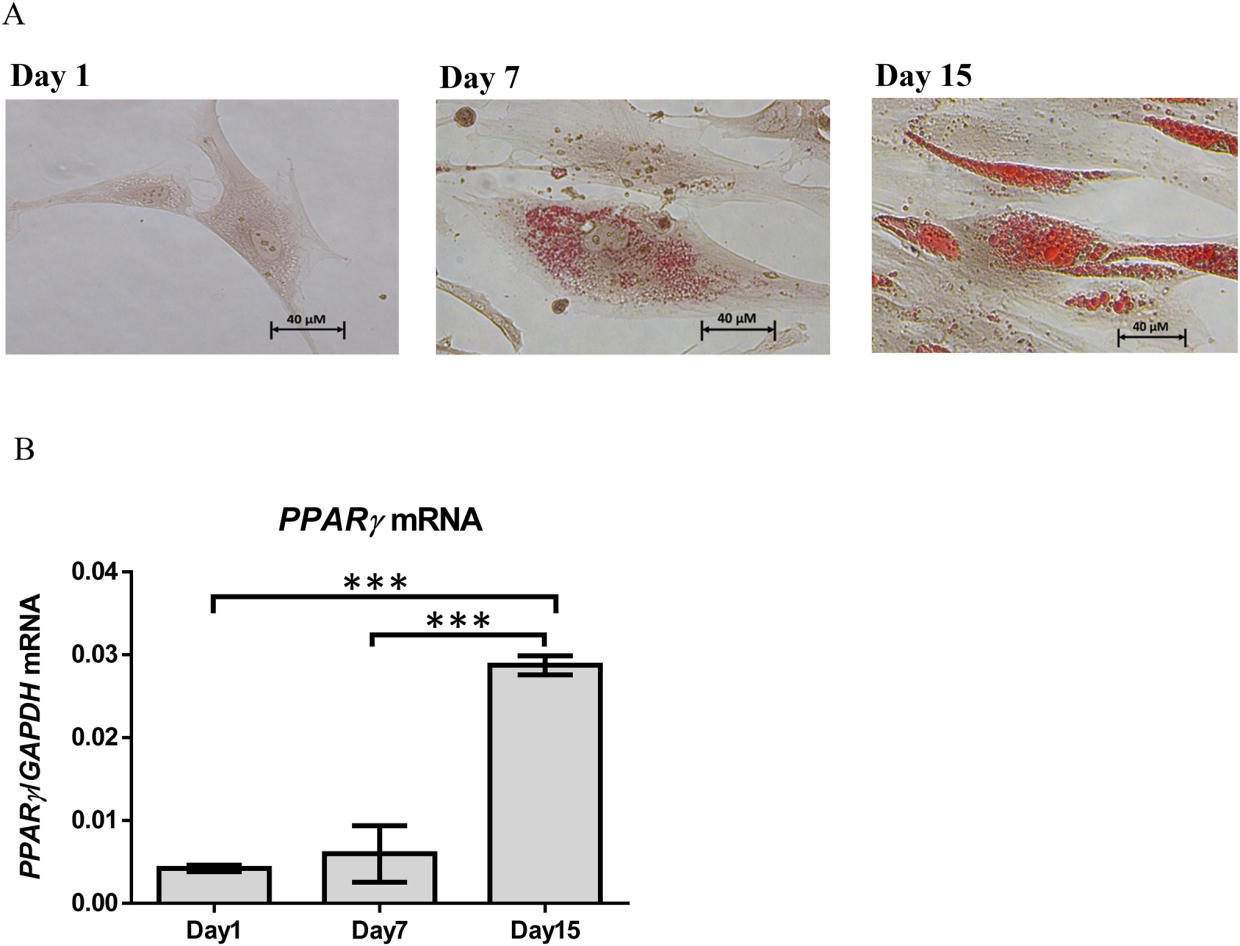
Adipocyte differentiation. (**A**) shows Oil Red O staining of human pre-adipocytes/adipocytes on day 1, day 7, and day 15. Red represents staining of lipid. (**B**) shows mean (± SEM) *PPAR-γ* mRNA expression normalized to *GAPDH* (a reference gene) in pre-adipocytes/adipocytes on day 1, day 7, and day 15, ***p < 0.001.

The study protocols of lipolysis and browning of human adipocytes were exempted by the Siriraj Institutional Review Board of the Faculty of Medicine Siriraj Hospital, Mahidol University (the protocol number 492/2559 (Exemption) and 451/2560 (Exemption), respectively). Human pre-adipocytes (Cell Applications, California, USA) were cultured with pre-adipocyte growth medium (Cell Applications, California, USA) supplemented with 1% penicillin/streptomycin (Corning, New York, USA) at 37 °C in the 5% CO_2_ humidified incubator. The medium was changed every other day until the human pre-adipocytes reached 100% confluent. Then, the cells were cultured with adipocyte differentiation medium (Cell Applications, California, USA) supplemented with 1% penicillin/streptomycin which was changed every 3 days for 15 days to induce differentiation. At the end of 15 days, the cells were differentiated into mature human adipocytes appearing with lipid droplets in the cells. Pre-adipocyte differentiation was confirmed by detecting lipid filling in adipocytes by Oil Red O staining shown in Fig. 5A and measuring mRNA expression of *PPAR-γ*, the adipocyte differentiation marker^51^, by real-time Polymerase Chain Reaction (PCR) shown in Fig. 5B, before starting experiments. The results showed that lipid droplets could not be detected in human pre-adipocytes on day 1; very small lipid droplets were detected in human pre-adipocytes on day 7; and many lipid droplets were detected in mature human adipocytes on day 15 suggestive of adipocyte maturation (Fig. 5A). Moreover, *PPAR-γ* mRNA was significantly higher on day 15 when compared with day 7 and day 1 (Fig. 5B).

### The lipolysis experiment

Fifty-five thousand human pre-adipocytes were cultured and induced differentiation on the round cover glass placed in 12 well plates. Mature human adipocytes were starved in the serum-free medium without phenol red (Corning, New York, USA) for 2 h before starting the experiment. Then, the cells were treated with 0.01% dimethyl sulfoxide (DMSO) as vehicle, 10 µM isoproterenol as the positive control, and 0.1, 1, 10, 100, 1000, and 10,000 ng/ml CQ extract for 24 h. Each condition of the experiment was done in triplicate. Glycerol release in the medium was measured by the Lipolysis Colorimetric Assay Kit (Sigma-Aldrich, Missouri, USA). The range of glycerol detection was 0–10 nmole/μl. Reading absorbance of O.D. at 570 nm was performed by the Synergy HT Multi-Detection Microplate Reader (BioTek Instruments, Inc., Vermont, USA). The lipid content in adipocytes was measured by determining the fat area assay of Oil Red O staining of adipocytes per image. A previous study revealed that the fat area assay measured by the total area of fat droplets determined with Oil Red O staining per image (the actual fat levels determined with Oil Red O) was the accurate and quick method and had a very strong positive correlation (R = 0.8787, *p* < 0.0001) with fat accumulation in differentiated adipocytes^52,53^. After Oil Red O staining, the round cover glass was removed out of the culture plates, placed on glass slides, scanned for the whole slide by the ScanScope XT machine using 20X magnification. Then, the scanned images were measured for the total fat area by using the AxioVisionVR software Release 4.8.2 (Carl Zeiss AG, Oberkochen, Germany). Then, the glycerol release was analyzed by the glycerol concentrations in samples normalized by the total fat area so the data are presented as glycerol concentrations/total lipid.

### Browning of human white adipocytes

One-hundred thousand human pre-adipocytes were cultured and induced differentiation in 6 well plates. Mature human adipocytes were treated with 0.01% DMSO as vehicle, 10 µM isoproterenol, 3 µM pioglitazone, 10 µM isoproterenol plus 3 µM pioglitazone, 10 ng/ml CQ extract, 100 ng/ml CQ extract, 1000 ng/ml CQ extract, 3 µM pioglitazone plus 10 ng/ml CQ extract, 3 µM pioglitazone plus 100 ng/ml CQ extract, and 3 µM pioglitazone plus 1000 ng/ml CQ extract mixed with the serum-free medium for 7 days. The medium was changed every 3 days. Each condition of the experiment was done in triplicate. The cells were harvested to isolate RNA and kept at -70^◦^C until analysis.

### Real-time PCR analysis

*PPARγ*, *UCP1,* and *GAPDH* mRNA expressions were quantified by real-time PCR. Briefly, total RNA was extracted by the TRIzol reagent (Invitrogen, California, USA) following the manufacturer’s instruction. Complementary DNA (cDNA) was synthesized by reverse transcription of 1 mg of total RNA by the iScript cDNA Synthesis Kit (Bio-Rad, California, USA). Real-time PCR was carried out using the reagents and protocol contained in the biotechrabbit QPCR Green Master Mix LROX, 2x (Biotechrabbit, Berlin, Germany). *GAPDH* was used as the reference gene because its expression was not changed under various experimental studies in human adipocytes^54^. The sequences of the primers were designed by the authors and blasted to check primer specificity by using nucleotide sequences published in PubMed database. All primer sequences were published in our previous study^55^ as follows:

*PPARγ*^55^ Forward-5′ AAAGTGCAATCAAAGTGGAGCC 3′.

Reverse-5′ CAAACCTGATGGCATTATGAGAC 3′.

*UCP1*^55^ Forward-5′ GCTCCAGGTCCAAGGTGAATG 3′.

Reverse-5′ CAATGAATACTGCCACTCCTCCA 3′.

*GAPDH*^55^ Forward -5′ GCCAGCCGAGCCACATC 3′.

Reverse -5′ GCTCCTGGAAGATGGTGATGG 3′.

The PCR amplification was performed by the CFX96 Real-Time PCR Detection System (Bio-Rad, California, USA) under the following conditions: UDG treatment at 50 °C for 2 min, Taq DNA polymerase activation at 95 °C for 3 min, 40 cycles of DNA denaturing at 95 °C for 15 s, annealing at 57 °C for 30 s for *UCP1* and *GAPDH* and 54 °C for 30 s for *PPAR-γ*, and extension at 65 °C for 30 s. Each reaction was done in duplicate. A negative control was performed by no template control. A comparative procedure of quantification was calculated by the 2^–ΔCT^ method.

### Statistical analysis

Test of normality was performed by the Kolmogorov–Smirnov test. Data were presented as mean ± SEM. The two-way mixed-design ANOVA which is the ANOVA with one repeated-measures factor and one between-groups factor was used to investigate the effect of treatment over time. Comparisons of 2 dependent groups were done by Paired Student’s t-test. Comparisons more than 2 dependent groups were performed by repeated-measured ANOVA after checking for homogeneity of variance followed by the Fishers Least Significant Difference (LSD) analysis. Comparisons of more than 2 independent groups were performed by one-way ANOVA after checking for homogeneity of variance followed by the LSD analysis. A statistical significance was considered at *p*-value < 0.05.

## Data Availability

The datasets generated during and/or analyzed during the current study are available from the corresponding author on reasonable request.
